# Design and Validation of a Self-care Evaluation Instrument to Prevent Diabetic Foot*

**DOI:** 10.17533/udea.iee.v40n2e06

**Published:** 2022-09-16

**Authors:** Eugenia del Pilar Herrera Guerra, Lili Rosa Bautista Arellanos

**Affiliations:** 1 Nurse, Ph.D. Professor, Universidad de Córdoba (Colombia). Email: edherrera@correo.unicordoba.edu.co. Universidad de Córdoba Colombia edherrera@correo.unicordoba.edu.co; 2 Statistician, Professor, Universidad de Córdoba(Colombia). Email: lbautistaarellano72@correo.unicordoba.edu.co Universidad de Córdoba Colombia lbautistaarellano72@correo.unicordoba.edu.co

**Keywords:** diabetic foot, primary prevention, self-care, nursing theory, psychometry., pie diabético, prevención primaria, autocuidado, teoría de enfermería, psicometría., pé diabético, prevenção primária, autocuidado, teoria de enfermagem, psicometria

## Abstract

**Objective.:**

This work sought to design and validate a self-care instrument to prevent diabetic foot in Colombian adults with diabetes.

**Methods.:**

Psychometric study in which an instrument was designed to measure self-care to prevent diabetic foot according to the Medium Range Theory of Self-care in chronic diseases. With a sample of 230 people with type-2 diabetes, construct validity was determined through exploratory and confirmatory factor analysis. Internal consistency was calculated with Cronbach's alpha coefficient.

**Results.:**

Favorable evidence of construct validity was obtained with a model consisting of three scales: self-care maintenance with a three-factor structure (accumulated variance 43%), α = 0.7, with good fit (𝜒2= 64.698, p = 0.001; RMSEA = 0.066; RMSSR = 0.071; CFI = 0.936, NNFI = 0.910). Monitoring of self-care with presence of symptoms a two-factor structure was found, α = 0.950, with good fit (𝜒2 = 266.837, p = 0.000; RMSEA = 0.321; RMSSR = 0.057; CFI = 0.848; NNFI = 0.789); and without symptoms, a single-factor structure (cumulative variance 84%), α = 0.9, acceptable fit (𝜒2= 377.327, p < 0.001; RMSEA = 0.355; RMSSR = 0.073; CFI = 0.832; NNFI = 0.764). And self-care management with two-factor structure (cumulative variance 53.7%) α = 0.7, with good fit (𝜒2 = 14.317, p = 0.014; RMSEA = 0.144; RMSSR = 0.063; CFI = 0.905; NNFI = 0.809).

**Conclusions.:**

The resulting instrument has adequate psychometric properties, consistent with the theoretical model of self-care in chronic diseases. Its use is recommended to evaluate self-care to prevent diabetic foot in populations similar to the study population.

## Resumen

**Objetivo.:**

Diseñar y validar un instrumento de autocuidado para prevenir el pie diabético en adultos colombianos con diabetes.

**Métodos.:**

Estudio psicométrico en el que se diseñó un instrumento para la evaluación del autocuidado para prevención el pie según la Teoría de Mediano Rango del Autocuidado en enfermedades crónicas. Con una muestra de 230 personas con diabetes tipo 2, se determinó la validez de constructo mediante análisis factorial exploratorio y confirmatorio. La consis tencia interna se calculó con el coeficiente alfa de Cronbach.

**Resultados.:**

Se obtuvo evidencia favorable de validez de constructo con un modelo formado por tres escalas: mantenimiento del autocuidado con una estructura de tres factores (varianza acumulada 43 %), α = 0,7 con buen ajuste (𝜒2 = 64.698, *p =* 0,001; RMSEA = 0.066; SRMR = 0.071; CFI = 0.936, NNFI = 0.910); monitoreo del autocuidado con la presencia de síntomas halló una estructura bifactorial (varianza acumulada 74.8 %) α = 0.950, con buen ajuste (𝜒2 = 266.83, *p* < 0,0001; RMSEA = 0.321; SRMR = 0.057; CFI = 0.848; NNFI = 0.789) y sin presencia de síntomas con una estructura unifactorial (varianza acumulada 84 %) α = 0,9 con ajuste aceptable (𝜒2 = 377.327, *p* < 0.01; RMSEA = 0.355; SRMR = 0.073; CFI = 0,832; NNFI = 0.764) y la tercera escala gestión del autocuidado con una estructura bifactorial (varianza acumulada 53.7%) α = 0.7, con buen ajuste (𝜒2= 14.317, *p* = 014; RMSEA = 0.144; SRMR = 0.063; CFI = 0.905; NNFI = 0.809).

**Conclusión.:**

El instrumento resultante posee propiedades psicométricas adecuadas, coherente con el modelo teórico del autocuidado en enfermedades crónicas. Se recomienda su uso para la evaluación del autocuidado para la prevención del pie diabético en poblaciones similares a la población de estudio.

## Resumo

**Objetivo.:**

Desenhar e validar o instrumento de autocuidado para prevenir o pé diabético em adultos colombianos com diabetes.

**Métodos.:**

Estudo psicométrico no qual foi elaborado um instrumento para avaliação do autocuidado para prevenção do pé de acordo com a Teoria de Médio Alcance do Autocuidado em doenças crônicas. Com uma amostra de 230 pessoas com diabetes tipo 2, a validade de construto foi determinada por análise fatorial exploratória e confirmatória. A consistência interna foi calculada pelo coeficiente alfa de Cronbach.

**Resultados.:**

Evidência favorável de validade de construto foi obtida com um modelo composto por três escalas: manutenção do autocuidado com estrutura de três fatores (variância cumulativa 43%), α = 0.7 com bom ajuste ( 𝜒 2= 64.698, p = 0,001; RMSEA = 0.066 , SRMR = 0.071, CFI = 0.936, NNFI = 0.910); monitoramento do autocuidado com a presença de sintomas, foi encontrada uma estrutura bifatorial (variância acumulada 74,8%) α = 0.950, com bom ajuste ( 𝜒 2 = 266.83, p < 0.0001; RMSEA = 0.321; SRMR = 0.057; CFI = 0.848; NNFI = 0.789) e sem a presença de sintomas com estrutura unifatorial (variância cumulativa 84%) α = 0.9 com ajuste aceitável ( 𝜒 2 = 377.327, p <0.01; RMSEA = 0.355; SRMR = 0.073; CFI = 0.832; NNFI = 0.764) e a terceira escala de gestão do autocuidado com estrutura bifatorial (variância cumulativa 53.7%) α = 0.7, com bom ajuste ( 𝜒 2= 14.317, p = 014; RMSEA = 0.144; SRMR = 0.063; CFI = 0.905; NNFI = 0.809).

**Conclusão.:**

O instrumento resultante apresenta propriedades psicométricas adequadas, condizentes com o modelo teórico de autocuidado em doenças crônicas. Seu uso é recomendado para avaliação do autocuidado para prevenção do pé diabético em populações semelhantes à população do estudo.

## Introduction

Diabetes mellitus (DM) is one of the biggest problems for health systems in Latin America. In Colombia, three of every 100 inhabitants has DM and it is one of the main causes of death among people between 30 and 70 years of age.^1^ It is estimated that between 15% and 20% of diabetics Will have foot ulcers during the course of its evolution and of these 30% will suffer amputation.^2^ Multiple risk factors are known to contribute to foot ulcers, hence, emphasis is placed on the importance of screening and classifying foot injuries and specific education to prevent diabetic foot.^3^ Thereby, the person with DM must enter a structured educational program from the moment of diagnosis to learn about the disease and become empowered to achieve and maintain adherence to the treatment and achieve self-care.^4^

It is known that self-care is carried out in both healthy and diseased states; however, self-care could have a different meaning in patients with chronic disease, given that it requires a set of behaviors to control the disease process, diminish the burden of symptoms, and improve survival.^5^ Therefore, health professionals and researchers need valid and reliable instruments that permit evaluating self-care in people with chronic diseases and prove the effectiveness of interventions focused on promoting self-care in this group of people. 

Design of valid and reliable instruments to evaluate self-care in people with chronic disease is useful for the nursing discipline because they permit conducting research that provides useful scientific evidence for research and for the professional practice. According with Riegel *et al.,*^5^,^6^ the design of instruments based on middle range theories constitute empirical indicators that permit testing the practical utility of the theory through research and allows the empirical evidence provided by studies to be used to ratify, modify, or refine theories. 

Currently, instruments exist to evaluate self-care in people with chronic diseases in overall manner 6 and specifically for different chronic diseases, like heart failure,^7^ coronary heart disease,^8^ chronic obstructive pulmonary disease,^9^ arterial hypertension,^10^ which are validated in Colombia^11^ and in DM.^12^ All these instruments were designed with the theoretical bases of the Middle Range Theory (MRT) of Self-care in chronic diseases.^13^

The Self-Care of Diabetes Inventory (SCODI) permits measuring self-care in people with DM. It consists of four scales: maintenance of self-care with three factors that evaluate exercise behaviors that promote health, behaviors of health promotion and disease prevention. The scale of monitoring self-care with two factors: listening to the body and recognizing symptoms. The scale of management of self-care that includes two factors: autonomous behaviors and consultative behaviors, and the scale of confidence in self-care that reflects the degree of confidence patients have on their capacity to carry out a specific task related with self-care.^12^,^13^ The SCODI includes only one item related with maintenance of self-care of the feet (Do you take care of your feet (wash them, dries the skin, applies creams, and wears socks or orthopedic stockings?) and a single item related with monitoring self-care (Do you control the state of your feet daily to check for injuries, reddening, or blisters?). It does not include behaviors related with management of self-care to prevent diabetic foot. 

Consequently, the study observed lack of information to evaluate specifically behaviors of self-care to prevent diabetic foot. Due to this, the aim of this study was to design and validate an instrument that permits evaluating self-care to prevent diabetic foot in people with DM2. 

## Methods

A psychometric study was conducted.^14^ The study was carried out by following the phases proposed by LoBiondo and Habers 15, which are described hereinafter.

### Phase I. Theoretical definition of the construct to be measured

A theoretical, methodological and empirical review of the self-care construct was performed and of the scientific evidence of foot care to prevent diabetic foot. The MRT of self-care in chronic diseases defines the self-care construct as a process to maintain health through health promotion practices and disease management. It has three concepts: maintenance of self-care, monitoring self-care, and management of self-care.^13^ The objective of maintenance is to maintain health and prevent exacerbations of symptoms; the objective of monitoring is the recognition that bodily change has occurred, monitoring or listening to the body, and the objective of management of self-care is that responding to symptoms implies an evaluation of changes in physical and emotional signs and symptoms to determine if action is necessary. Monitoring of personal care is the link between maintenance of self-care and management of self-care. For example, people with DM2 perform monitoring activities, like measuring blood sugar and early detection of signs and symptoms of changes in blood glucose; likewise, they must understand its severity to take measures before the situation worsens.^5^,^13^ Within this context, the study reviewed behaviors of self-care to prevent diabetic foot. The Colombian guidelines for the prevention, diagnosis, and treatment of diabetic foot proposes five basic elements to prevent diabetic foot: daily direct observation of the feet; maintaining the skin clean, fresh, and moisturized; wearing with daily change of special prevention stockings: footwear, adapted to the type of feet and immediate direct and trustworthy communication and among patients and their relatives and the management staff.^2^ These recommendations were kept in mind to formulate the items of the instrument.

### Phase II. Formulation of the instrument’s items

The Instrument of Self-care for prevention of diabetic foot (ISPDF) was designed with the theoretical bases of the MRT of self-care in chronic diseases.^13^ Self-care behaviors identified in the literature permitted the construction of the items grouped into three scales to reflect the theory’s three central concepts: maintenance of self-care, monitoring self-care, and management of self-care. The scale of maintenance of self-care included the common recommendations given by health providers for the prevention of the diabetic foot related with daily care of the feet (maintaining the skin clean, fresh, and moisturized, wearing special stockings and footwear) and behaviors related with the disease (adherence to treatment regimen and monitoring and controlling diabetes). The self-care monitoring behaviors discussed in the literature were grouped into behaviors related with listening to the body, identifying the first manifestations of foot injuries, and monitoring signs and symptoms. The self-care management behaviors were grouped into autonomous self-care as actions to eliminate risk factors and consultative self-care, like direct and immediate communication upon the presence of symptoms of diabetic foot with the family and health staff.

### Phase III. Development of instructions for users and experts

During the design of the first version of the new instrument denominated ISPDF, the different phases of test creation proposed by Muñiz and Fonseca-Pedrero were followed.^16^ The purpose of the instrument was determined, assuming that the variable to be observed is self-care. Upon drafting the items, it was taken into account that they were not ambiguous, seeking to express a single idea per statement. The scale was constructed with 29 items, specifying the characteristics of the items and its Likert-type response format, according to the three dimensions to evaluate: maintenance of self-care (10 items) with five response options, where never is equal to 1 and always is equal to 5. Monitoring self-care (without symptoms 9 items, with symptoms 11 items) has five response options, where never is equal to 1 and always is equal to 5 and items 20 and 21 with 6 response options, where *I did not recognize the symptom* is equal to 0 and very quickly is equal to 5. The scale of management of self-care (8 items) with 5 response options, where never is equal to 1 and always is equal to 5.

### Phase IV. Validity and reliability tests of the Instrument

The apparent and content validity of the ISPDF was evaluated by six judges (nurses with PhD, clinical and psychometry experience), who evaluated the instrument based on three qualifying criteria: comprehension, clarity, and precision. Fleiss’ kappa index was calculated,^17^ which permitted determining agreement between observers correcting for chance. The results were interpreted as satisfactory those items obtaining values comprised between 0.61 and 0.80, recognized as substantial agreement. These same experts evaluated each of the items with the following criteria: “essential”, “useful but not essential”, and “not necessary”. With the data obtained, the content validity ratio and the content validity index of the whole instrument were calculated, following the modified Lawshe model,^18^ which establishes a value > 0.58 to consider an item as acceptable, independent of the number of evaluators.

To carry out the construct validity tests, there was a sample of 234 people with DM2 registered in diabetes control programs in four healthcare centers of the network of first level of care in Montería- Colombia, in 2019. To determine the sample size, a number > 200 and a rate > 5 subjects per variable were set, recommended for psychometric analyses, to offer Good guarantees in estimating the parameters, especially with models that include few variables with respect to that proposed by Gorsuch.^19^ According to the foregoing, from a population of 614 people registered in diabetes control programs, a sample was chosen of 234 participants calculated with 95% confidence level and 5% margin of error. The sample selection process was through convenience. The inclusion criteria were being older than 18 years, having a medical diagnosis of DM2 according to the criteria of the Clinical Practice Guideline for the diagnosis of DM2 in population over 18 years of age.^20^ The study excluded patients with high comorbidity, mental and/or sensory deficit.

A sociodemographic characteristics questionnaire developed by the researchers was applied to each of the participants together with the ISPDF instrument designed. To ensure the quality of the data, logistic and operational aspects were taken into account, such as training for the application of the instrument, providing a place free of interference, giving information to participants before administering the instrument, and adopting the comprehensibility and completeness criteria, seeking to know that the participants understood the indications the instrument contemplates and verifying that all the items were respectively filled out and correctly completing the study database.

The data were analyzed in the SPSS statistical program version 22.0. Initially, an analysis was performed of the descriptors (mean, standard deviation, asymmetry, kurtosis, and item-total corrected correlation coefficient), expecting to obtain calculations of the asymmetry and kurtosis indices, between ± 1.96 in the normality test.^20^ To assess the adequacy of the sample size and the correlation among variables, the Kaiser-Meyer-Olkin test was used (≥ 0.6 is acceptable) and Bartlett's sphericity test (*p* < 0.05) prior to implementing the factorial analysis. The exploratory factorial analysis (EFA) was performed by using maximum likelihood as extraction method, with Oblimin rotation. An inflection point of 0.32 was taken as the minimum value of factor loading required to maintain each element extracted from the factorial analysis. The criterion to determine what items belong to the factor is the factor loading, which indicates the degree of relation between the item and the factor. The loads of all the elements must be > 0.30.^21^

For the Confirmatory Factor Analysis (CFA), the data were processed in the IBM SPSS Amos statistical package - version 26.0. The following goodness of fit indices were evaluated: the p value associated with the Chi-squared (X^2^) statistic, which tests the null model against the hypothesized or proposed one. Not resulting statistically significant (p > 0.05) can be interpreted as indicator of an adequate fit of the model to the data. The comparative fit index (CFI) was also included, which compares the improvement in the fit of the model in question with a null model to evaluate the degree of loss produced in the fit when changing from the model proposed to the null model) and the non-normed fit index (NNFI or TLI), which reflects the total proportion of information explained by a model; to accept the model proposed, the value of CFI, TLI must be ≥ 0.9. Among the indices based on the covariances, the work opted for the root mean square error of approximation (RMSEA) and the root mean square standardized residual (RMSSR), considered optimal when their values are 0.05 or less, and acceptable in the range from 0.05 to 0.08. Akaike's information criterion (AIC) is considered along with the PRATIO parsimony index as measurements of the relative quality of a statistical model because, given a set of candidate models for the data, the model with the best fit is that with the minimum value of these measures.(22) 

The internal consistency was evaluated by calculating Cronbach’s alpha (α) coefficient. The corrected element-total correlation was determined as corrected homogeneity coefficient; if < 0.2, it is eliminated. A value of α ≥ 0.7 is expected to consider that the ISPDF is reliable for use in research.^23^

The study was conducted according with Resolution 8430 of 1993^24^ and Legislation 911 of 2004.^25^ It had the University’s ethical endorsement and obtained approval from the healthcare institution where the participants were recruited to carry out the study. All the participants provided their written consent. 

## Results

### Apparent and content validity 

The 29 items of the preliminary version of the ISPDF were scored by the panel of experts (*n* = 6) with impact score > 1.5. Hence, these were adequate and retained. The study obtained Fleiss’ kappa index of 0.7 in comprehension, 0.8 in clarity, and 0.8 in precision, which was interpreted as substantial agreement. All the items were accepted (content validity ratio > 0.79); the content validity index was reported as satisfactory (0.9). 

### Construct validity

Of the sample of 234 people with DM2, most were women (57%); the mean age was 55 years; the majority reported low educational level (86%) and low income (89%), as shown in Table 1. The average time of being diagnosed with DM2 was four years. The results obtained in the descriptive analysis, based on asymmetry and kurtosis, provides information to detect data normality.

**Table 1:** Sociodemographic characteristics of the sample (*n* = 234)

Variable	Frequency	%
Sex		
Female	133	57
Male	101	43
Educational level		
Writes and reads	82	35
Primary	119	50.9
Secondary	18	7.7
Technical	9	3.8
Professional	6	2.6
Income		
< 1 CLMW	210	89.7
> 1 CLMW	24	10.3
Has health social security	234	100

CLMW: current legal minimum wage in Colombia, 2019

Maintenance of self-care. For the self-care maintenance scale, the internal consistency resulted acceptable with α = 0.70. Given the results of the corrected item-total correlation, there was no need to eliminate any item. The Kaiser-Meyer-Olkin (KMO) sample adequacy index was adequate to apply the EFA, which obtained a three-factor structure that explains 43% of the accumulated variance. The first factor was constituted by items 8, 9, and 10; the second factor by items 4, 5, 6, and 7; and the third factor by items 1, 2, and 3, with item 2 having the lowest communality.

In the CFA, the model suggested by the EFA had better results with respect to the single-factor model of the 10 items that conform the maintenance scale (𝜒2 = 64.698, p = 0.001; RMSEA = 0.066; RMSSR = 0.071; CFI = 0.936, NNFI = 0.910) being a good fit according to interpretation criteria of the fit indices, as shown in Table 2.

**Table 2:** EFA and CFA of the self-care maintenance scale of the ISPDF

EFA (KMO = 0.738; Chi squared: 547.233; df = 45; 𝑝 < 0.000)			
α =0.7 n=234		Factor loading	
10 items	Factor 1	Factor 2	Factor 3
1. Do you wash and dry your feet, especially between the toes?			0.577
2. Do you moisturize your feet with moisturizing cream to prevent dryness?			0.379
3. Do you take care that your feet do not stay wet for a long time?			0.552
4. Do you cut your nails straight, avoiding the use of sharp objects?		0.585	
5. Do you wear thick, seamless, non-pressure stockings without holes or special stockings for people with diabetes?		0.787	
6. Do you use good shoes, preferably those that when the sole is folded, stay rigid or have a personalized insole adapted to your feet?		0.655	
7. Do you take care of your feet from the cold and heat?		0.441	
8. Do you attend your medical and/or nursing check-up appointments?	0.842		
9. Do you follow the doctor’s and/or nurse’s recommendations for diabetes control?	0.743		
10. Do you comply with the treatment to control diabetes?	0.742		
% Variance by factor	21.122	17.886	4.403
% Accumulated variance	21.122	39.008	43.411

**Table 2:** (Continuation) EFA and CFA of the self-care maintenance scale of the ISPDF

CFA										
Models	Absolute Fit Measurements				Incremental fit measures		Parsimony fit measures			
	Chi squared	df	SRMSR	RMSEA	90%CI	CFI	NNFI	PRATIO	AIC
10-item single-factor model	261.019	35	0.000	0.153	0.166	0.148 - 0.186	0.559	0.433	0.778	301.019
Three-factor model suggested by the EFA	64.698	32	0.001	0.071	0.066	0.043 - 0.089	0.936	0.91	0.711	130.698

Extraction method: principal component analysis. Loads > 0.32 were accepted. Rotation method: Varimax normalization with Kaiser. CI: confidence interval: χ2: chi-squared; df: degrees of freedom; *significance p > 0.05; AIC: Akaike Index; NNFI: Non-Normed of Fit Index; CFI: Comparative Fit Index; RMSSR: Root Mean Standard Square Residual; RMSEA: Root Mean Square Error of Approximation.

Monitoring self-care. In the self-care monitoring scale conformed by 11 items, observations associated with item 19 (Keep a record of symptoms?) had zero variance, not providing information to the calculation of Cronbach’s alpha and the factorial analysis, which is why it was excluded from the analysis. Monitoring self-care can be evaluated in patients with and without symptoms of diabetic foot. The EFA was performed in the sample with symptoms of diabetic foot and in the sample without symptoms of diabetic foot. The results are presented ahead.

With diabetic foot symptoms. The KMO index had an adequate value to apply the factorial analysis. The EFA was conducted with 10 of the 11 items proposed in the scale, item 19 that was eliminated due to zero variance. The EFA results suggested a two-factor structure for which the factor loading of item 20 (How quickly did you recognize you had symptoms in the feet (reddening, blisters, injuries, burns, calluses, ingrown toe nails, fungus, infections, etc.?) did not exceed the minimum established, hence, it was eliminated, leaving a two-factor model that explains 59% of the accumulated variance, factor one conformed by items 11, 12, 13, 15, and 21 and factor two with items 14, 16, 17, and 18. The CFA results indicate a better fit for the two-factor model suggested by the EFA (𝜒2 = 266.837; 𝑝 = 0.000; RMSEA = 0.321; RMSSR = 0.057; CFI = 0.848; NNFI = 0.789). Internal consistency was quite good (α = 0.950). Table 3 shows the EFA and CFA results.

Without diabetic foot symptoms. The KMO value was Good to apply the EFA. Table 4 shows the EFA and CFA results. A single-factor structure was maintained conformed by eight items that explains 84% of the accumulated variance. The structure of the factor was constituted by items 11, 12, 13, 14,15, 16, 17, and 18, registering at least a communality of 0.725 for item 17. Note that item 21 should not be included, considering the nature of the question. Although not reaching the optimal values in the CFA for the fit indices, CFI, NNFI and RMSA, the RMSSR was within the acceptable ranges (𝜒2 = 377.327; 𝑝 = 0.000; RMSEA = 0.355; RMSSR = 0.073; CFI = 0.832; NNFI = 0.764) with respect to a single-factor structure. The internal consistency was quite good (α = 0.97). 

**Table 3:** EFA and CFA of the self-care monitoring scale of the ISPDF in people with diabetic foot symptoms

EFA (KMO = 0.769; Chi squared: 1594; df = 45; p < 0.000)								
α =0.95 n=91		Factor loading						
9 items		Factor 1			Factor 2			
11. Monitor injuries or evidence of future foot injuries daily?		0.991						
12. Pay attention to changes observed in feet?		0.985						
13. Observe the feet and spaces between the toes with a mirror or a magnifying glass?		0.955						
14. Watch for evidence of future foot injury (redness, swelling, calluses, etc.)?					0.978			
15. Check for foot injuries and infections (wounds, ulcers, ingrown nail infections, fungus, etc.)?		0.778						
16. Inform the health provider if you have a sensation of cutting pain in your feet, burning pain, numbness, sensation of needle-like pricking, pain in your legs that forces you to sit down?					0.941			
17. Closely monitor symptoms?					0.895			
18. Verify diabetic foot symptoms with the health provider?					0.971			
21. How quickly did you know that the symptom was due to diabetic foot?		0.368						
% Variance by factor		59.208			15.895			
% Accumulated variance		59.208			74.895			
CFA											
Models	Absolute Fit Measurements				Incremental fit measures	Parsimony fit measures			
	Chi squared	df		SRMSR	RMSEA	90%CI	CFI	NNFI	PRATIO	AIC
10-item single-factor model (without item 19)	820.301	35	0.000	0.132	0.499	0.470 - 0.529	0.517	0.379	0.778	865.87
Two-factor model suggested by the EFA	266.837	26	0.000	0.057	0.321	0.287 - 0.356	0.848	0.789	0.722	304.837

Source: own authorship. Extraction method: principal component analysis. Loads > 0.32 were accepted. Rotation method: Varimax normalization with Kaiser. CI: confidence interval: (𝜒2: chi-squared; df: degrees of freedom; *significance p > 0.05; AIC: Akaike Index; NNFI: Non-Normed of Fit Index; CFI: Comparative Fit Index; RMSSR: Root Mean Standard Square Residual; RMSEA: Root Mean Square Error of Approximation.

**Table 4:** EFA and CFA of the self-care monitoring scale of the ISPDF in people without diabetic foot symptoms

EFA (KMO = 0.850; Chi squared: 2097.34; df = 28; p < 0.000)					
α = 0.977 n=143		Factor loading			
8 items		Factor 1			
11. Monitor injuries or evidence of future foot injuries daily?		0.994			
12. Pay attention to changes observed in the feet?		0.994			
13. Observe the feet and spaces between the toes with a mirror or magnifying glass?		0.965			
14. Watch for evidence of future foot injury (redness, swelling, calluses, etc.)?		0.885			
15. Check for foot injuries and infections (wounds, ulcers, ingrown nail infections, fungus, etc.)?		0.859			
16. Inform the health provider if you have a sensation of cutting pain in your feet, burning pain, numbness, sensation of needle-like pricking, pain in your legs that forces you to sit down?		0.790			
17. Closely monitor symptoms?		0.852			
18. Verify diabetic foot symptoms with the health provider?		0.810			
% Variance by factor		84.412			
% Accumulated variance		84.412			
CFA					
Models	Absolute Fit Measurements				Incremental fit measures		
	Chi squared	df		SRMSR	RMSEA	90%CI	AIC
Single-factor model suggested by the EFA	377.327	20	0.000	0.073	0.355	0.324 - 0.386	409.327

Source: own authorship. Extraction method: principal component analysis. Loads > 0.32 were accepted. Rotation method: Varimax normalization with Kaiser. CI: confidence interval: χ2: chi-squared; df: degrees of freedom; *significance 𝑝 > 0.05; AIC: Akaike Index; NNFI: Non-Normed of Fit Index; CFI: Comparative Fit Index; RMSSR: Root Mean Standard Square Residual; RMSEA: Root Mean Square Error of Approximation.

Management of self-care. In the self-care management scale, the reliability analysis indicated that the internal consistency increased if item 25 was eliminated (Take medications to control diabetes?), given that it was correlated negatively with the other items, going from having Cronbach’s alpha from 0.608 to 0.667. The KMO permitted applying the EFA. A two-factor structure was obtained, which explains 53.7% of the accumulated variance, with acceptable internal consistency (α = 0.732) and fit (𝜒2 = 14.317, p= 0.014; RMSEA = 0.144; RMSSR = 0.063; CFI = 0.905; NNFI = 0.809); the results are shown in Table 5.

**Table 5:** EFA and CFA of the self-care management scale of the ISPDF

EFA (KMO = 0.610; Chi squared: 260.91; df = 21; p< 0.000)									
α =0.73 n=234					Factor loading				
7 items					Factor 1			Factor 2	
22. Eliminate factors that are injuring the feet (dryness, signs of pressure, moisture, etc.)?					0.464				
23. Perform cleaning and disinfection of foot injuries?					0.960				
24. Perform glycemic control (Glucometer)?					0.942				
26. Consult with health care provider for guidance?								0.742	
27. Ask a relative or friend for advice?								0.639	
28. Consult with the doctor immediately?								0.774	
29. Evaluate if the treatment improved the symptoms?								0.368	
% Variance by facto					26.470			27.278	
% Accumulated variance					26.470			53.749	
CFA									
Models	Absolute Fit Measurements				Incremental fit measures				Parsimony fit measures	
	Chi squared	df	p	SRMSR	RMSEA	90%CI	CFI	NNFI	PRATIO	AIC
7-item single-factor model (without item 25)	177.022	14	0.000	0.185	0.360	0.313 - 0.408	0.346	0.020	0.667	205.022
7-item two-factor model suggested by the EFA	14.317	5	0.014	0.063	0.144	0.059 - 0.234	0.905	0.809	0.500	34.317

Source: own authorship. Extraction method: principal component analysis. Loads > 0.32 were accepted. Rotation method: Varimax normalization with Kaiser. CI: confidence interval: χ2: chi-squared; df: degrees of freedom; *significance p> 0.05; AIC: Akaike Index; NNFI: Non-Normed of Fit Index; CFI: Comparative Fit Index; RMSSR: Root Mean Standard Square Residual; RMSEA: Root Mean Square Error of Approximation.

**Figure 1:**
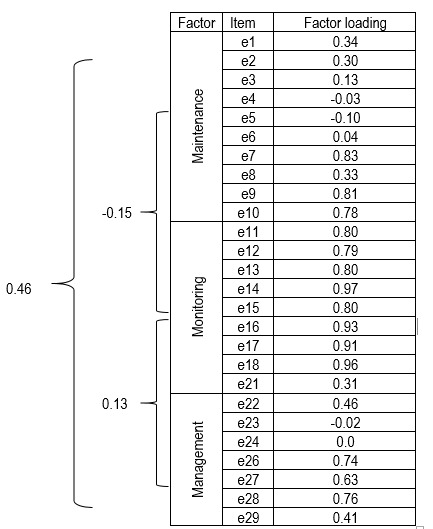
Structural diagram of the confirmed model and the factor loadings

Bearing in mind the results of reliability, of the EFA and CFA, the ISPDF was finally comprised of 26 items distributed into three scales: maintenance of self-care conformed by 10 items, monitoring of symptoms with 8 or 9 items with or without diabetic foot symptoms, respectively, and management of self-care with 7 items. The factor structure and the correlations obtained among the variables and the items from each of the scales are shown in Figure 1.

The following presents the scoring algorithm of the ISPDF, which was carried out by bearing in mind the theoretical-conceptual foundations associated with the measurement model that indicates that the instruments developed with the MRT od self-care in chronic diseases must follow a scoring algorithm that can be used to calculate the answers with standardized scores. The three scales have a standardized score range from 0 to 100. A higher score means better self-care. Each of the scales must be calculated separately, never globally. For interpretation, it is necessary to transform the score from each scale into a standardized score ranging between 0 and 100 using the following formula: actual raw score - lowest possible raw score/ possible raw score range by 100.^26^

To calculate the scores of each of the ISPDF scales, first add the total score to obtain the actual raw score. In the self-care maintenance scale (10 items), the lowest possible raw score is 10 and the highest is 50; the possible raw score range is 40. Monitoring self-care, with 8 or 9 items, depends on the lack or presence of symptoms; the lowest possible raw score is 8 or 9 and the highest possible is 40 or 45, thereby, the possible raw score range is 32 or 36, respectively. In the self-care management scale with 7 items, the lowest possible raw score is 7 and the highest possible is 40 and the possible score range is 33. The final version of the instrument designed is included as annex at the end of this article. 

## Discussion

The aim of this study was to design a new instrument that permits evaluating self-care to prevent diabetic foot and evaluate its validity and reliability psychometric properties. The ISPDF was designed, ensuring the theoretical base of the instrument in an MRT that describes, explains, and predicts self-care in chronic diseases.

Now, this is not the first study that applies the MRT of self-care in chronic diseases on the design of instruments that permit measuring self-care; this theory has been widely used in the design and validation of instruments with good results.^7^^-^12 Studies conducted reflect the empirical adequacy of said theory in the study of the self-care construct that comprises three core concepts, that is, maintenance of self-care, monitoring self-care, and management of self-care that represent the theoretical dimensions of self-care in chronic diseases. 

The exploratory factorial analysis for the ISPDF reported a three-factor structure in the variable for maintenance of self-care; the factor loadings ranged between 0.36 and 0.96. The goodness-of-fit indices for this model were statistically significant with acceptable fit. The correlations estimated between the maintenance and monitoring variables were -0.15; between maintenance and management 0.46, and between monitoring and management 0.13. The correlations in the maintenance scale with 10 items ranged between -0.3 and 0.83, indicating low, moderate and high correlations.

In the ISPDF, the scale for maintenance of self-care measures the behaviors destined to daily care of the feet grouped into three factors that promote health, prevent the disease, and help to maintain the disease stable through adherence to DM treatment. This result has been found in other psychometric studies of instruments that assess self-care developed with the theoretical model of the MRT of self-care in chronic diseases, which show that maintenance of self-care is a multifactor scale.^27^

The EFA for the ISPDF in the variable for self-care monitoring reported a two-factor structure in people with diabetic foot symptoms; the factor loadings ranged between 0.36 and 0.99. The goodness-of-fit indices for this two-factor model were statistically significant with acceptable fit. The correlation estimated among the factors was 0.71 and the correlation with most of the items was > 0.8, indicating moderate and high correlations. This study identified a two-factor model for the scale of self-care monitoring or perception of symptoms, similar to that proposed in the theoretical model that includes two factors: listening to the body and recognition of symptoms.^12^,^13^

For the self-care management scale, the EFA reported a two-factor structure; the factor loadings ranged between 0.36 and 0.96. The goodness-of-fit indices for this two-factor model were all statistically significant with acceptable fit. The correlations estimated among the factors were 0.71 and correlations for the factors with the items ranged between -0.2 and 0.76, indicating low and moderate correlations. The two-factor model is similar to that proposed in the theoretical model that includes two factors: autonomous behaviors and consultative behaviors that characterize the behaviors used by people with DM2 to control their symptoms.^12^,^13^

It must be highlighted that the EFA for the ISPDF was guided by the MRT of self-care in chronic diseases. This prior theory permitted proposing hypothesis on the number of factors and of the pattern expected. The validity results obtained confirm the theoretical hypotheses that support the self-care construct in chronic diseases, demonstrating that the three scales of the ISPDF permit measuring self-care. These results are consistent with previous studies conducted in people with diabetes applying the Self-Care of Diabetes Inventory,^12^ the Self-Care of Heart Failure Index,(7) the Self-Care of Coronary Heart Disease Inventory,^8^ the Self-Care in Chronic Obstructive Pulmonary Disease Inventory 9, and the self-care of hypertension inventory 10, which demonstrates the empirical adequacy of the MRT of self-care in chronic diseases in the design of new instruments, like the ISPDF.

The differences found with respect to other studios regarding those found in this study are related with the dimensionality of the scale of self-care maintenance; prior psychometric studies, like the Self-Care of Heart Failure Index, 7 have demonstrated that behaviors of self-care maintenance comprise two dimensions or factors: consultation behaviors and dietetic behaviors. The Self-Care of Diabetes Inventory^12^ comprises four dimensions labeled as: exercise behaviors that promote health (Factor 1), disease prevention behaviors (Factor 2); health promotion behaviors (Factor 3), and behaviors related with the disease (Factor 4). 

Reliability measured as internal consistency of the scale of self-care maintenance (α = 0.7), monitoring self-care (α = 0.9), and management of self-care (α = 0.7), was adequate in the sample studied. According with the MRT of self-care of chronic diseases, self-care behaviors reflect a sequence, i.e., most patients first dominate self-care maintenance and then construct experience in monitoring self-care and management of self-care. That is, the three concepts are closely related, illustrating that they are related behaviors from the same general construct; hence, effective performance of self-care encompasses the three behaviors, which must be measured separately and never globally.^5^

This study had some limitations, the sample was recruited in a municipality of the country and, although representing different ages and sexes and the sample size was that suggested for psychometric studies (n > 200), it may not be stated that it is representative of the whole Colombian population with DM2. Other psychometric tests of the ISPDF should be performed in different zones of the country and with larger samples. This study did not prove the capacity to detect changes in the construct over time; this requires experimental studies with interventions that promote self-care to permit evaluating the effects of the changes in the self-care behavior and contribute to refining the theory to create sound evidence-based knowledge.

The impact the study could have for the population diagnosed with DM2 and for the nursing discipline lies mainly on the need to include in the nursing evaluation empirical indicators derived from the MRT of self-care in chronic diseases, which permit identifying where the person is having problems to carry out the self-care behaviors; necessary information to perform specific and adapted interventions centered on improving self-care processes (maintenance, monitoring, and management). Studies reveal that high levels of self-care improve metabolic control,^28^ reduce hospitalizations,^29^ complications related with DM2,^30^ and improve health-related quality of life in people with DM2.^31^

In conclusion, the ISPDF is a valid and reliable instrument that permits measuring self-care to prevent diabetic foot in people with DM2 in population similar to the conditions in this study, based on the MRT of self-care in chronic diseases. The instrument is comprised of 26 items distributed into three independent scales: self-care maintenance, monitoring self-care, and management of self-care that showed good psychometric properties in a Colombian population. Its use is recommended for research.

## Appendix. Instrument to evaluate self-care to prevent diabetic foot

All answers are confidential.

Think of how you have felt during the last month while you fill out this questionnaire.

**Table 10:** SECTION A. Listed below are behaviors that people with Type 2 Diabetes Mellitus use to help themselves to maintain foot care. How often or routinely do you do the following? (Check a number)

Maintenance of self-care	Never		Sometimes		Always
1. Do you wash and dry your feet, especially between the toes?	1	2	3	4	5
2. Do you moisturize your feet with moisturizing cream to prevent dryness?	1	2	3	4	5
3. Do you take care that your feet do not stay wet for a long time?	1	2	3	4	5
4. Do you cut your nails straight, avoiding the use of sharp objects?	1	2	3	4	5
5. Do you wear thick, seamless, non-pressure stockings without holes or special stockings for people with diabetes?	1	2	3	4	5
6. Do you use good shoes, preferably those that when the sole is folded, stay rigid or have a personalized insole adapted to your feet?	1	2	3	4	5
7. Do you take care of your feet from the cold and heat?	1	2	3	4	5
8. Do you attend your medical and/or nursing check-up appointments?	1	2	3	4	5
9. Do you follow the doctor’s and/or nurse’s recommendations for diabetes control?	1	2	3	4	5
10. Do you comply with the treatment to control diabetes?	1	2	3	4	5

**Table 11:** SECTION B. The following lists the changes people with Type 2 Diabetes Mellitus tend to monitor in their feet. How often do you do the following? (Check a number)

Monitoring of self-care	Never		Sometimes		Always
11. Monitor injuries or evidence of future foot injuries daily?	1	2	3	4	5
12. Pay attention to changes observed in the feet?	1	2	3	4	5
13. Observe the feet and spaces between the toes with a mirror or magnifying glass?	1	2	3	4	5
14. Watch for evidence of future foot injury (redness, swelling, calluses, etc.)?	1	2	3	4	5
15. Check for foot injuries and infections (wounds, ulcers, ingrown nail infections, fungus, etc.)?	1	2	3	4	5
16. Inform the health provider if you have a sensation of cutting pain in your feet, burning pain, numbness, sensation of needle-like pricking, pain in your legs that forces you to sit down?	1	2	3	4	5
17. Closely monitor symptoms?	1	2	3	4	5
18. Verify diabetic foot symptoms with the health provider?	1	2	3	4	5

**Table 12:** Continuation

19. How quickly did you know that the symptom was due to diabetic foot?	I have not had symptoms	I did not recognize the symptom	Not quickly	Somewhat quickly	Quickly	Very quickly
	N/A	1	2	3	4	5

**Table 13:** SECTION C. The following lists the behaviors people with Type 2 Diabetes Mellitus tend to use to control their symptoms of diabetic foot. How likely are you to try the following actions? (Check a number)

Management of self-care	Unlikely		Somewhat likely		Very likely
20. Eliminate factors that are injuring the feet (dryness, signs of pressure, moisture, etc.)?	1	2	3	4	5
21. Perform cleaning and disinfection of foot injuries?	1	2	3	4	5
22. Perform glycemic control (Glucometer)?	1	2	3	4	5
23. Consult with health care provider for guidance?	1	2	3	4	5
24. Ask a relative or friend for advice?	1	2	3	4	5
25. Consult with the doctor immediately?	1	2	3	4	5
26. Evaluate if the treatment improved the symptoms?	1	2	3	4	5
